# Integrated approach to functional analysis of an *ERBB2* variant of unknown significance detected by a cancer gene panel test

**DOI:** 10.1007/s13402-021-00656-3

**Published:** 2022-01-08

**Authors:** Yohei Harada, Akemi Sato, Mitsugu Araki, Shigeyuki Matsumoto, Yuta Isaka, Yukari Sagae, Tomonori Abe, Yasuko Aoyagi, Eisaburo Sueoka, Yasushi Okuno, Shinya Kimura, Naoko Sueoka-Aragane

**Affiliations:** 1 Division of Hematology, Respiratory Medicine and Oncology, Department of Internal Medicine, Faculty of Medicine, Saga University, 5-1-1 Nabeshima, Saga, 849-8501 Japan; 2 Department of Clinical Laboratory Medicine, Faculty of Medicine, Saga University, 5-1-1 Nabeshima, Saga, 849-8501 Japan; 3 Graduate School of Medicine, Kyoto University, 53 Shogoin-Kawaharacho, Sakyo-ku, Kyoto, 606-8507 Japan; 4 Medical Sciences Innovation Hub Program, RIKEN Cluster for Science, Technology and Innovation Hub, 1-7-22 Suehiro-cho, Tsurumi-ku, Yokohama City, Kanagawa 230-0045 Japan; 5 AI-driven Drug Discovery Collaborative Unit, RIKEN Center for Computational Science (R-CCS), HPC- and AI-driven Drug Development Platform Division, 7-1-26 Minatojimaminamimachi Chuo-ku, Kobe City, Hyogo 650-0047 Japan; 6 Department of Precision Cancer Medicine, Center for Innovative Cancer Treatment, Tokyo Medical and Dental University, 1-5-45 Yushima, Bunkyo-ku, Tokyo, 113-8510 Japan

**Keywords:** *ERBB2*, Variants of unknown significance, Extracellular domain, Functional analysis, Molecular dynamics simulation

## Abstract

**Purpose:**

Dealing with variants of unknown significance (VUS) is an important issue in the clinical application of NGS-based cancer gene panel tests. We detected a novel *ERBB2* extracellular domain VUS, c.1157A > G p.(E401G), in a cancer gene panel test. Since the mechanisms of activation by *ERBB2* extracellular domain (ECD) variants are not fully understood, we aimed to clarify those mechanisms and the biological functions of *ERBB2* E401G.

**Methods:**

*ERBB2* E401G was selected as VUS for analysis because multiple software tools predicted its pathogenicity. We prepared *ERBB2* expression vectors with the E401G variant as well as vectors with S310F and E321G, which are known to be activating mutations. On the basis of wild-type *ERBB2* or mutant *ERBB2* expression in cell lines without *ERBB2* amplification or variants, we evaluated the phosphorylation of human epidermal growth factor receptor 2 and related proteins, and investigated with molecular dynamics (MD) simulation the mechanisms conferred by the variants. The biological effects of *ERBB2* E401G were also investigated, both in vitro and in vivo.

**Results:**

We found that *ERBB2* E401G enhances C-terminal phosphorylation in a way similar to S310F. MD simulation analysis revealed that these variants maintain the stability of the EGFR-HER2 heterodimer in a ligand-independent manner. Moreover, *ERBB2* E401G-transduced cells showed an increased invasive capacity in vitro and an increased tumor growth capacity in vivo.

**Conclusion:**

Our results provide important information on the activating mechanisms of *ERBB2* extracellular domain (ECD) variants and illustrate a model workflow integrating wet and dry bench processes for the analysis of VUS detected with cancer gene panel tests.

**Supplementary Information:**

The online version contains supplementary material available at 10.1007/s13402-021-00656-3.

## Introduction

The use of comprehensive genomic panel tests based on next-generation sequencing (NGS) has increased in clinical practice for cancer patients [1]. In principle, it can guide therapeutic strategies through the detection of targetable/druggable molecular alterations specific to each individual patient with cancer. However, the number of cancer patients who can receive treatment based on the results of sequence data has been reported to be approximately 10% among those tested with NGS [2, 3]. To improve this clinical situation, we need to develop better methods for the analysis and interpretation of molecular profiles obtained with NGS. One issue to be resolved is how to effectively deal with variants of unknown significance (VUS). The Association for Molecular Pathology (AMP) recommends a four-tiered system based on criteria using several sources, such as published clinical and experimental results, population databases, and cancer-specific variant databases [4]. Among the four tiers, VUS is defined as tier III when convincing evidence of cancer association is not found in a published database or when the variant has not been reported at a significant allele frequency in general or subpopulation-specific databases. Such a VUS is not regarded as a targetable variant, although the possibility that it is amenable to treatment cannot be ruled out.

Recently, a patient with a cancer of unknown primary (CUP) origin was referred to us. This patient had a VUS corresponding to the criteria mentioned above, which was detected using a NGS panel test. The variant was located in extracellular domain (ECD) III of *ERBB2 (HER2/Neu)*, which encodes human epidermal growth factor receptor (HER) 2 protein, c.1157A > G p.(E401G). *ERBB2* gene alterations such as *ERBB2* amplification and activating mutations are promising target alterations, as has been shown in some multi-histology basket trials: the MyPathway trial [5] and the SUMMIT trial [6]. Most variants functionally analyzed were located in the kinase domain, such as a G776YVMA insertion in exon 20 and V777L, which constitutively phosphorylates and activates HER2 [7–10], but functional analyses of ECD variants have been limited [11, 12]. As far as we are aware, even with hot spot ECD mutations, such as S310F, the underlying mechanisms of activation are not adequately understood [13].

*ERBB2* E401G is a novel ECD variant that has not been registered in any major database, but its appearance in multiple in silico algorithms for predicting variant pathogenicity suggests that it has a deleterious effect. Therefore, we investigated the biological effect of *ERBB2* E401G and mechanisms related to its effects by using in vitro and animal model experiments, as well as in silico molecular dynamics (MD) simulation analysis. Here, we present a workflow model of functional analysis applied to a VUS detected in an actual case, and we illustrate the use of this model for seeking appropriate evaluation and analysis by integrating wet and dry bench processes.

## Methods

### Cell lines and reagents

Human lung cancer cell lines NCI-H460, A549 and NCI-H2170, and mouse fibroblast cell line NIH3T3 were purchased from the American Type Culture Collection (ATCC). NCI-460, A549 and H2170 cells were cultured in RPMI-1640 medium supplemented with 10% FBS. NIH3T3 cells were cultured in DMEM medium supplemented with 10% FBS.

### Droplet digital PCR for detection of mutant *ERBB2*

Droplet digital PCR (ddPCR™) mutation detection assays were performed using a Bio-Rad QX200 Droplet Generator™ and Droplet Reader (Bio-Rad Laboratories, Inc. Hercules, CA, USA) according to the manufacturer’s protocol. For detecting *ERBB2* c.1157A > G p.(E401G) and wild type *ERBB2* c.1157A (pE401), custom probes labeled with FAM and HEX, respectively, designed on Bio-Rad’s Digital Assay Site were used. Analysis was performed using Bio-Rad QuantaSoft software.

### Plasmid constructs and transfection

Myc-DDK-tagged ORF clones of human cDNAs encoding full-length HER2 (RC212583) and empty vector (PS100001) were obtained from Origene (Rockville, MD, USA). E401G, S310F, E321G and D845A mutations were introduced by using a QuikChange Site-Directed Mutagenesis Kit (Agilent Technologies, Inc., Santa Clara, CA, USA) and were verified by direct sequencing. Each plasmid DNA was transfected into cells with Lipofectamine (Thermo Fisher Scientific, Waltham, MA, USA) according to the manufacturer’s protocol.

### Western blot analysis

Whole cell lysates were extracted using lysis buffer (50 mM Tris-HCL pH 8.0, 50 mM Tris-HCL pH 8.0, 150 mM NaCl, 5 mM MgCl2, 1% TritonX-100, 0.1% sodium dodecyl sulfate, 0.5% sodium deoxycholate, 40 mM sodium fluoride, and 1 mM sodium orthovanadate) containing phosphatase inhibitors, protease inhibitors and 20 mM DTT, as reported previously [14]. Western blot analysis was carried out by conventional methods using the following primary antibodies: anti-HER2, anti-phospho(P)-HER2 (Tyr1221/1222), anti-EGFR, anti-P-EGFR (Tyr1068), anti-HER3, anti-P-HER3 (Tyr1289), anti-Akt, anti-P-Akt (Ser473), anti-p44/p42 MAPK, anti-P-p44/p42 MAPK (Thr202/Tyr204), anti-β-actin (used as a loading control) (Cell Signaling Technology, Danvers, MA, USA) and anti-FLAG® M2 (Sigma-Aldrich, St. Louis, MO, USA). As secondary antibodies HRP-conjugated anti-mouse or anti-rabbit IgG (Cell Signaling Technology, Danvers, MA, USA) were used. Proteins were detected using a C-Digit® imaging system (LI-COR Biosciences, Lincoln, NE, USA) and were visualized using Image Studio™ for C-Digit®. A dimerization assay was performed as reported previously [11]. The cell lysate preparation and subsequent procedures were the same as indicated above, expect that DTT (reducing agent) was not included in the lysis buffer.

### Structure modeling of HER2-HER2 and EGFR-HER2 dimers

Initial structural data on the extracellular domains of wild-type HER2 and EGFR were obtained from the Protein Data Bank [PDB codes: 3WLW (the “back-to-head” HER2 homodimer) and 3NJP (the “back-to-back” EGFR homodimer bound to EGF), respectively] (Fig. S2a). For modeling the “back-to-head” EGFR-HER2 heterodimer, the “back-to-back” EGFR-HER2 heterodimer, and the “back-to-back” HER2 homodimer, the HER2 (EGFR) subunit in the crystal structures was replaced with the EGFR (HER2) subunit after structural alignment using HER2 residues 268–338 and EGFR residues 240–309 in domain II [15]. A detailed modeling protocol is described below. In the crystal structure of the “back-to-head” HER2 homodimer (PDB codes: 3WLW), domain II in subunit A interacts closely with subunit B, while that in subunit B is exposed to the solvent (Fig. S2a). To model the “back-to-head” EGFR-HER2 heterodimer, subunit A of the HER2-HER2 homodimer was replaced with the EGFR subunit (subunit A of the EGFR-EGFR homodimer). The “back-to-back” EGFR-HER2 and HER2-HER2 dimers were modeled on the basis of the crystal structure of the “back-to-back” EGFR homodimer (PDB codes: 3NJP). To model the “back-to-back” EGFR-HER2 heterodimer, subunit A of the EGFR-EGFR homodimer was replaced with the HER2 subunit (subunit A of the HER2-HER2 homodimer). To model the “back-to-back” HER2-HER2 homodimer, subunits A and B of the EGFR-EGFR homodimer were replaced with the HER2 subunit (subunit A of the HER2-HER2 homodimer). The initial structure for ligand-free EGFR was prepared by removing EGF from EGF-bound EGFR. Structures of disordered loops were modeled using the Structure Preparation module in the Molecular Operating Environment (MOE) program v. 2016.08 (Chemical Computing Group Inc., 1010 Sherbrooke St. West, Suite #910, Montreal, QC, Canada, H3A 2R7, 2016). The N- and C-termini of the protein models were capped with acetyl and N-methyl groups, respectively. The dominant protonation state at pH 7.0 was assigned for titratable residues. A S310F or E401G mutation on HER2 was introduced into the wild-type structure by using MOE.

### Molecular dynamics (MD) simulation

MD simulations were performed using the GROMACS 2019.1 program [16]. The Amber ff99SB-ILDN force field [17] was used for protein and ions, and TIP3P [18] was used to model water molecules. From 75,000 to 91,000 water molecules were placed around each protein model with an encompassing distance of 8 Å. 150 mM sodium and chloride ions were introduced into the simulation box to neutralize the system. Electrostatic interactions were calculated by using the particle mesh Ewald (PME) method [19] with a cutoff radius of 10 Å. Van der Waals interactions were cut off at 10 Å. Virtual sites for hydrogen atoms were used to allow for a time step of 4 fs [20]. The P-LINCS algorithm was employed to constrain all bond lengths [21]. After energy-minimization, each system was equilibrated for 100 ps in a constant number of molecules, volume and temperature (NVT) ensemble and run for 100 ps in a constant number of molecules, pressure and temperature (NPT) ensemble, with positional restraints applied on protein heavy atoms. The temperature was maintained at 310 K by stochastic velocity rescaling [22] and a Parrinello-Rahman barostat was used to maintain the pressure at 1 bar [23]. The temperature and pressure time constants were set to 0.1 and 2 ps, respectively. For each of the “back-to-head” EGFR-HER2 WT heterodimer, “back-to-head” HER2 WT-HER2 WT homodimer, “back-to-back” EGFR-HER2 WT heterodimer, “back-to-back” EGFR/EGF-HER2 WT heterodimer and “back-to-back” HER2 WT-HER2 WT homodimer, three independent 1-μs production runs were performed with different velocities. For the “back-to-back” EGFR/EGF-HER2, EGFR-HER2 and HER2-HER2 dimers, five independent 1-μs production runs were performed for each of wild-type HER2 and its S310F or E401G mutant. The buried area in each dimer was calculated by counting the contributions from domains I–III with a probe radius of 2.5 Å, using the High Throughput Molecular Dynamics (HTMD) environment 1.14.0 [24].

### In vitro cell invasion and proliferation assays

An in vitro cell invasion assay was performed using Corning® BioCoat™ Matrigel® Invasion Chambers with an 8 μm pore size (Corning Life sciences, Corning, NY, USA). At 24 h after transfection into H460 cells of empty vector, wild type (WT), E401G and S310F *ERBB2* containing vectors, cells were counted and 5 × 10^4^ cells were seeded into respective Boyden chambers. Cell invasion and migration were induced by FBS 24 h after seeding (48 h after transfection), after which the membranes were stained. The numbers of invading cells and migrating cells were counted in each 16 mm^2^ area. The invasion rate was determined by the following formula (100 × mean number of cells invading through the matrigel insert membrane / mean number of cells migrating through the control insert membrane) according to the manufacturer’s protocol. For analysis of cell proliferation in vitro, at 24 h after seeding H460 cells onto a 6-well plate, transfection of empty vector, wild type, E401G and S310F *ERBB2* containing vectors was conducted. At 24, 48, 72 and 96 h after transfection, the number of viable cells was counted using a TC20™ Automated Cell Counter (Bio-Rad Laboratories, Inc. Hercules, CA, USA).

### Evaluation of tumor growth in vivo

Immunodeficient Balb/c Rag-2−/− Jak3−/− (BRJ) mice, which lack mature T and B lymphocytes and natural killer cells [25], were provided by Seiji Okada (Kumamoto University, Kumamoto, Japan). The mice were housed under pathogen-free conditions in animal facilities at Saga University according to institutional guidelines. Stably transfected H460 cells with wild type *ERBB2* (H460-WT), E401G *ERBB2* (H460-E401G) and vector alone (H460-emp) were established with a selection procedure using G418 (Takara Bio USA, Inc., Mountain View, CA, USA) after transfection. H460-emp, H460-WT and H460 E401G cells (5 × 10^5^ each) were injected into the right dorsal flanks of 8-week-old female BRJ mice. Tumor sizes were measured twice per week using calipers, and tumor volumes were calculated using the empirical formula V = 1/2 × [(the shortest diameter)^2^ × (the longest diameter)]. On the 21st day after inoculation, the mice were sacrificed and the tumors were photographed.

### Statistical analysis

Data are expressed as mean with standard deviation (SD). Differences between two groups were tested using Student’s t test. Differences among three or more groups (each *ERBB2* mutant vs. wild-type *ERBB2*) were tested using one-way ANOVA with the Dunnet multiple comparisons test. For all comparisons, *p* < 0.05 was considered statistically significant. All calculations were performed using JMP Pro 14.2.0 (SAS Institute Inc., Cary, NC, USA).

## Results

### Detection of *ERBB2* E401G VUS in a patient with CUP

**Fig. 1:**
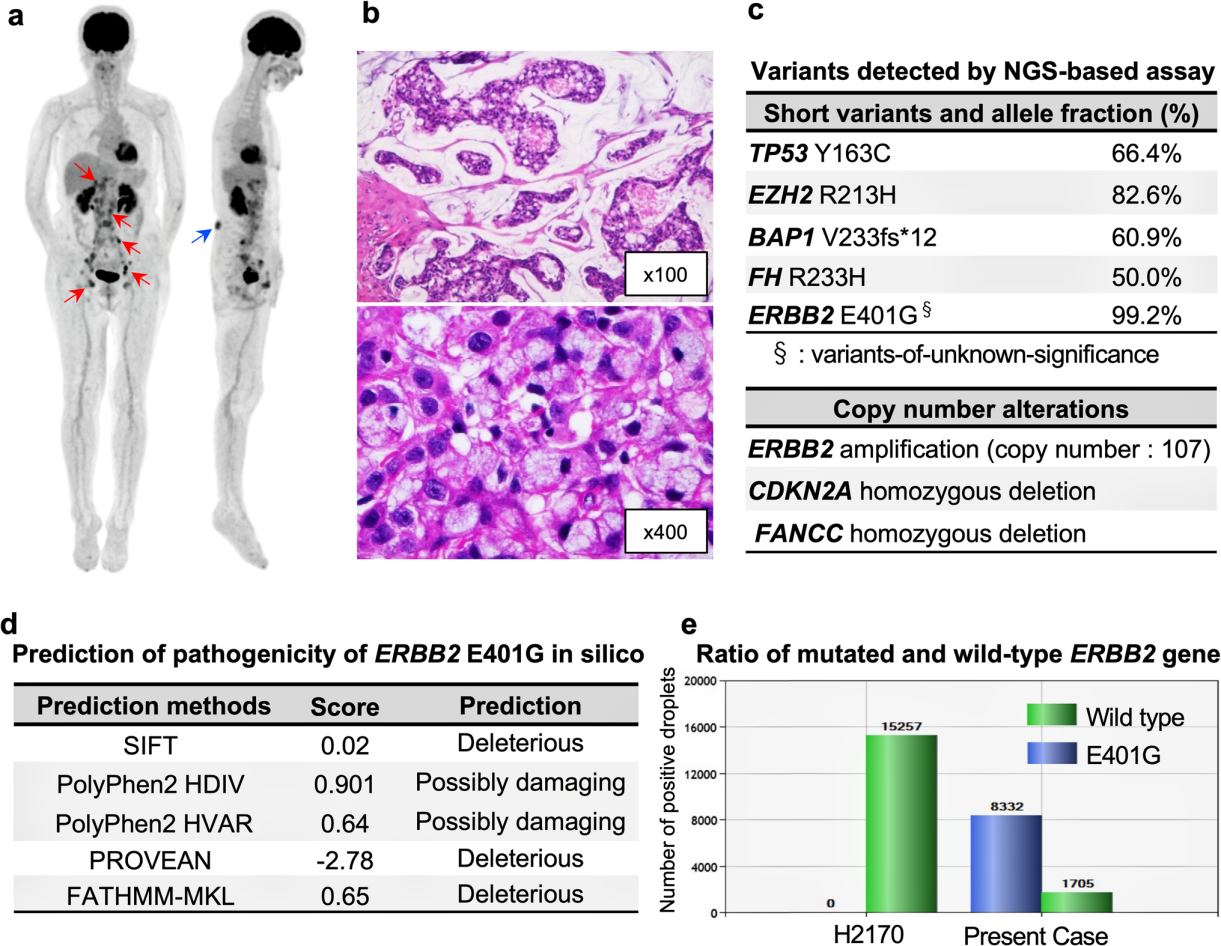
Clinical features and detected variants in the CUP patient. **a** Positron emission tomography/computed tomography before treatment. Red arrows indicate lesions of abdominal and inguinal lymphadenopathy and the blue arrow indicates a subcutaneous mass in the lower back. **b** Hematoxylin and eosin histology of the excisional biopsy of an inguinal lymph node, showing adenocarcinoma producing a large amount of mucus (upper figure). Some cancer cells exhibited a signet-ring-like-morphology (lower figure). **c** Table showing detected variants considered to be pathogenic. *ERBB2* E401G is a variant of unknown significance, but is included in this table because some in silico prediction tools suggested its pathogenicity. Copy number alterations detected in this case are listed; gene amplifications interpreted as equivocal (four copies) are not shown. **d** Results of in silico pathogenicity prediction. The cut-off value of each prediction method was as follows: SIFT (< 0.05: deleterious), PolyPhen2 (0.446 <, ≤ 0.908: Possibly Damaging, 0.908 <: Probably Damaging), PROVEAN (≤ −2.5: deleterious), FATHMM-MKL (0.5 <: deleterious). **e** Mutation detection assay by Droplet Digital PCR (ddPCR™) using genomic DNA extracted from a formalin-fixed, paraffin-embedded tumor tissue sample obtained from the patient or from H2170 cells. H2170 cells (wild type *ERBB2* amplification-positive) were used as a negative control

A 67-year-old Japanese woman, previous healthy, presented with right inguinal pain with no family history of cancer. Fluorodeoxyglucose (FDG)-positron emission tomography with CT showed increased FDG accumulation in the lower back subcutaneous mass and also in the hepatic hilum, para-aortic, iliac, and inguinal lymphadenopathy (Fig. 1a). Pathological examination of tissue from excisional biopsy of an inguinal lymph node revealed mucinous adenocarcinoma (Fig. 1b) with immunohistochemistry (IHC) positive for cytokeratin (CK) 7, CK20 and GATA3, and negative for CK5/6, CDX2 and p63. After additional examination, we diagnosed her with CUP. To find potential therapeutic targets, we performed a NGS-based multiplex gene assay using FoundationOne®CDx, by which 324 genes can be sequenced and concurrently examined for rearrangements of selected human solid cancer genes using the biopsy specimen. We found that *ERBB2* gene amplification (*ERBB2* copy number of 107) was accompanied by a missense variant, E401G, with an allele fraction of 99.2% (Fig. 1c). This *ERBB2* E401G variant has not been registered in the major variant databases COSMIC, ClinVar, the 1000 Genomes Project Database, ExAC and dbSNP at the time of this writing, but multiple computational tools, i.e., SIFT [26], PolyPhen-2 [27], PROVEAN [28] and FATHMM-MKL [29] supported a deleterious effect of *ERBB2* E401G on the encoded gene product. (Fig. 1d). The allele fraction of *ERBB2* E401G was unusually high, but as it was not found in the patient’s germline DNA it was considered to be a somatic mutation. Fluorescence in situ hybridization (FISH) confirmed the coexistence of *ERBB2* amplification and droplet digital PCR revealed that *ERBB2* E401G amplification dominated (Fig. 1e), which could explain the high allele fraction of *ERBB2* E401G. On basis of the *ERBB2* amplification, the patient was enrolled into the JUPITER trial (jRCT2031180150) [30], a basket trial of trastuzumab and pertuzumab for *ERBB2*-amplified solid tumors.

### *ERBB2* E401G has functional properties similar to those of S310F

To examine the functional properties of *ERBB2* E401G, an ECD III variant, we evaluated two types of mechanisms of activation of ECD variants previously reported: formation of disulfide-linked dimers and elevation of C-terminal phosphorylation [11]. To evaluate these mechanisms, we conducted transient transfection of H460 cells (human lung cancer cell line without variants or amplification of *ERBB* family genes) and NIH3T3 cells (mouse non-cancer fibroblast cell line) with an empty vector (empty) or a vector containing *ERBB2* wild type (WT) or one of four *ERBB2* variants. The *ERBB2* variants included three ECD variants, E321G, E401G or S310F, and a kinase domain inactivating variant of *ERBB2* D845A. The expression levels of HER2 and FLAG proteins were comparable between *ERBB2* WT and *ERBB2* variants (Fig. S1).

**Fig. 2:**
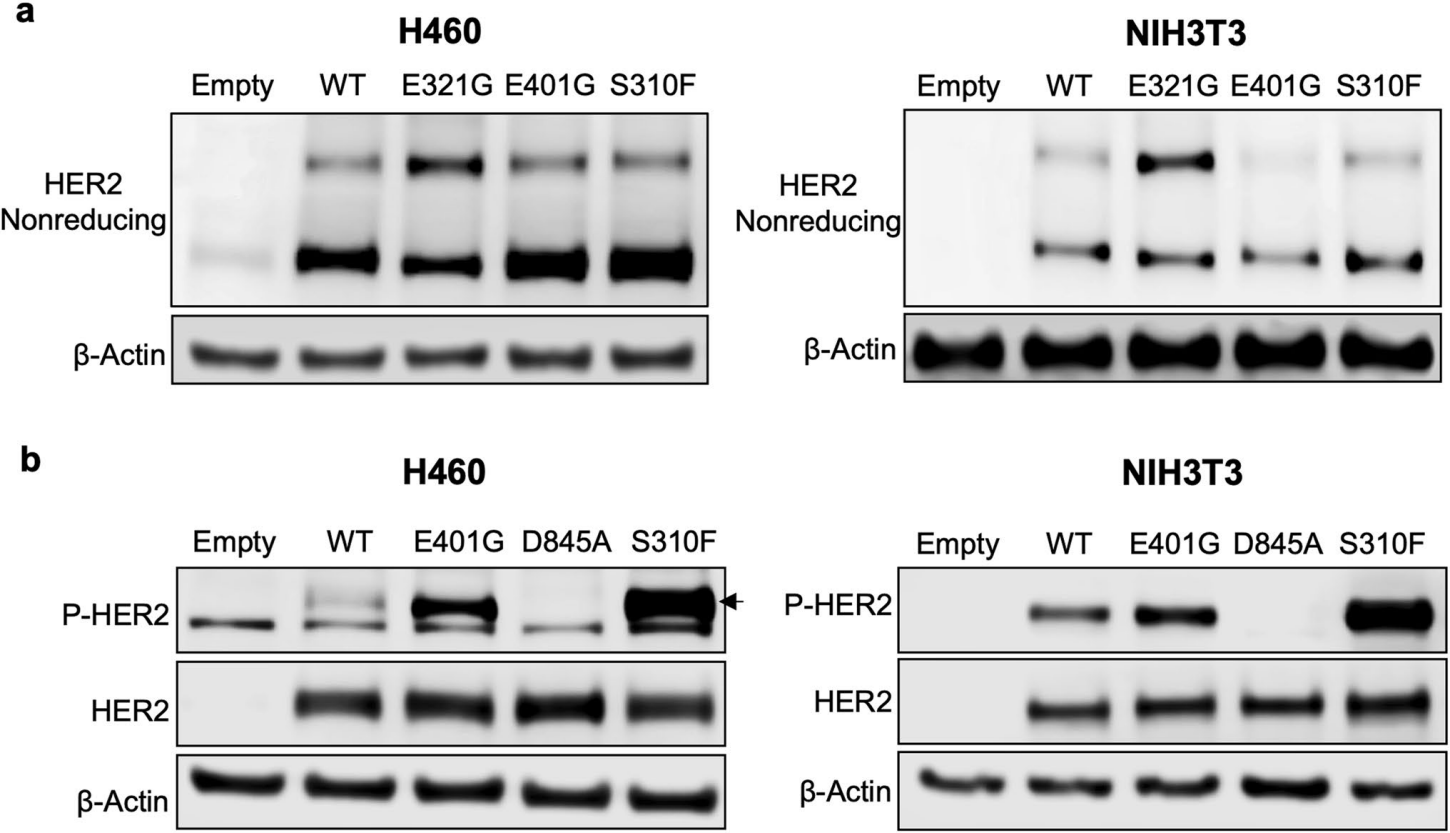
Formation of disulfide-linked HER2 dimers and C-terminal phosphorylation of HER2 in *ERBB2* E401G expressing cells. **a** Western blots under non-reducing conditions in H460 cells and NIH3T3 cells are shown. Both cell lines are expressing E401G as well as S310F, E321G or wild type *ERBB2*. E321G is a positive control mutant which can form disulfide-linked HER2 dimers. **b** Phosphorylation status of HER2 in H460 cells and NIH3T3 cells expressing E401G, S310F, D845A or wild type *ERBB2*. An *ERBB2* kinase-inactive mutant, D845A, was used as a negative control. Transfected H460 and NIH3T3 cells were grown under serum-starved conditions and exposed for three hours to serum-containing medium, after which whole-cell lysates were extracted. Empty, empty vector; WT, wild type; P-, phosphorylated

First, we examined whether E401G can form disulfide-linked dimers using SDS/PAGE under non-reducing conditions (for preserving disulfide bonds) and Western blotting. Compared with cells expressing *ERBB2* WT, H460 cells expressing *ERBB2* E321G (a positive control variant forming disulfide-linked dimers) and NIH3T3 cells showed robust increases in high-molecular weight bands consisting of HER2 dimers (Fig. 2a). On the other hand, *ERBB2* E401G- and S310F-expressing cells did not show increases in HER2 dimers (Fig. 2a), suggesting that *ERBB2* E401G is not relevant to the formation of disulfide-linked dimers.

Next, we analyzed C-terminal phosphorylation of HER2 using conventional SDS/PAGE and Western blotting. Compared with cells expressing *ERBB2* WT, cells expressing *ERBB2* S310F (a positive control variant elevating C-terminal phosphorylation) showed robust elevation of C-terminal phosphorylation (Fig. 2b). Cells expressing *ERBB2* E401G also showed increased phosphorylation of HER2, although the trend was less pronounced than with S310F (Fig. 2b). These results suggest that the mechanisms of *ERBB2* E401G activation are similar to those of S310F.

### Identification of potential dimerization partners of HER2 E401G protein

**Fig. 3:**
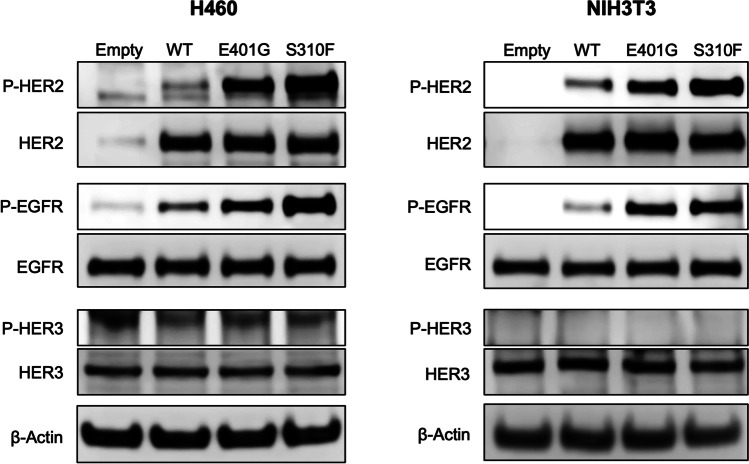
Phosphorylation of HER2 and EGFR in cells expressing *ERBB2* E401G. Transiently transfected H460 and NIH3T3 cells were grown under serum-starved conditions and exposed for three hours to serum-containing medium, after which whole-cell lysates were extracted, followed by Western blotting. Empty, empty vector; WT, wild type; P-, phosphorylated

C-terminal phosphorylation of HER family proteins is caused by dimerization followed by trans-autophosphorylation, in which one receptor subunit of the dimer phosphorylates the other [31, 32]. Among the HER family proteins, EGFR, HER2 and HER3 are considered to play critical roles in oncogenesis [33], but the role of HER4 in oncogenesis remains unclear. A previous study showed no significant role of HER4 expression in breast cancer survival [34]. To examine which HER2 family member may serve as the most important dimerization partner for activating the HER2 E401G mutant, we analyzed the phosphorylation of transfected HER2, EGFR and HER3 in H460 and NIH3T3 cells. We found that cells expressing *ERBB2* E401G or S310F exhibited increased phosphorylation levels of HER2 and EGFR, to a greater extent than cells expressing *ERBB2* WT (Fig. 3). In contrast, no differences in HER3 phosphorylation were observed (Fig. 3). These results suggest that the major dimerization partner of HER2 E401G and S310F is either HER2 itself or EGFR or both.

### HER2 p.(E401G) stabilizes ligand-free EGFR HER2 heterodimer

To confirm whether HER2 homodimers or EGFR-HER2 heterodimers are more relevant to the mechanisms of *ERBB2* E401G and S310F activation, we analyzed HER-family dimers using microsecond-timescale MD simulations. With regard to the crystal structure, most of the HER-family dimers form a symmetric complex, called the “back-to-back” model [15] (Fig. S2a). In contrast, there are some reports indicating that HER2 homodimers form an asymmetric complex via domain II and domain IV, called the “back-to-head” model [35, 36] (Fig. S2a). To assess which model is plausible for HER family dimers, we initially performed MD simulations starting from these two models. The results suggested that the overall structures of both EGFR-HER2 heterodimers and HER2 homodimers are more stably maintained in the “back-to-back” model than in the “back-to-head” model (Fig. S2b). On the basis of these simulation results, we adopted the symmetric “back-to-back” model for subsequent analyses.

**Fig. 4:**
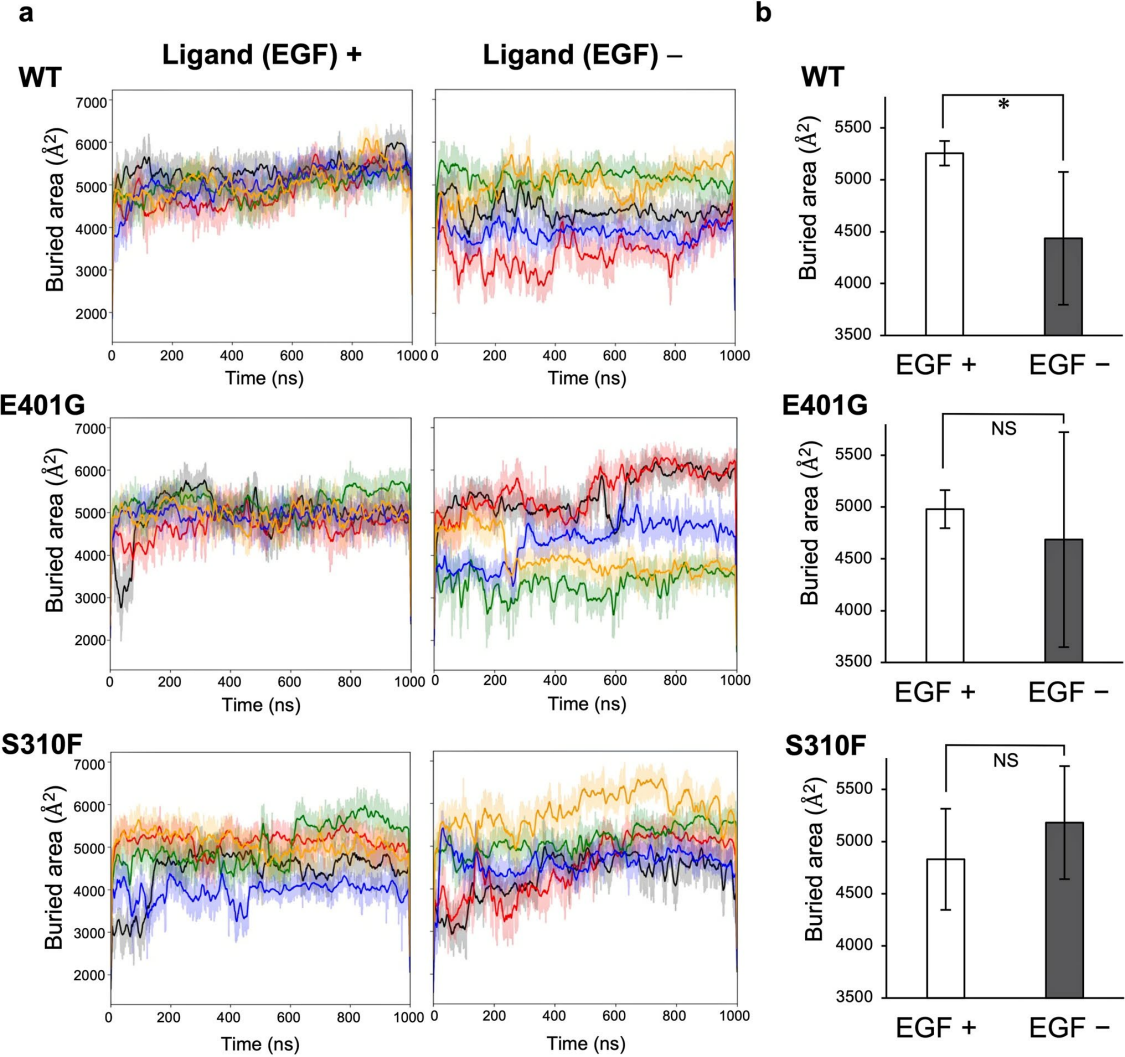
*ERBB2* E401G and S310F mutants maintain the stability of ligand-free EGFR-HER2 heterodimer in MD simulation. **a** Surface area buried in the dimer interface during five independent simulations of 1000 ns (black, red, green, blue and orange) of the EGF-bound (left) or EGF-unbound (right) form. Time-dependent transition of the buried area is plotted with thin lines along with a 10 ns window average (thick lines). **b** Buried area averaged across trajectories of 500–1000 ns extracted from the five simulations. The difference between EGF-bound and EGF-unbound forms was evaluated using a one-sided Student’s *t* test (*p < 0.05; NS, not significant). The dimer interfaces of HER2 E401G and S310F mutants tended to be stably maintained even in the absence of EGF

**Fig. 5:**
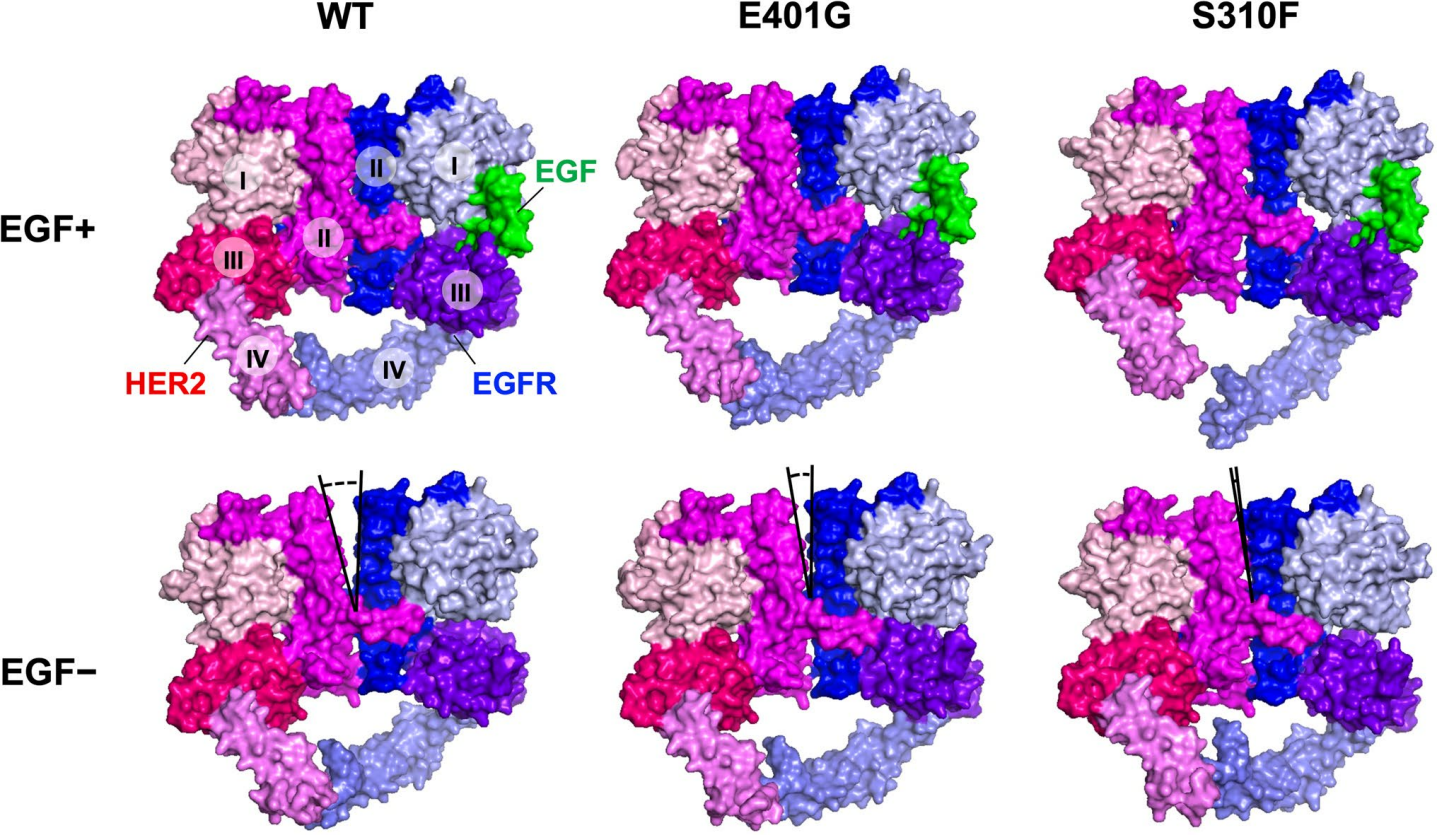
MD simulation structures of the HER2-EGFR heterodimer in the EGF-bound (upper) or EGF-unbound (lower) forms. The mean heterodimer structure was generated by trajectories of 500–1000 ns extracted from five independent simulations of 1000 ns, and is shown by a surface model. While a remarkable gap is observed in the dimer interface between HER2 WT and EGF-free EGFR, as indicated by a V-shaped outline, the gap tends to be closed in heterodimers with HER2 E401G and S310F mutants

In a previous simulation study, the dimer interfaces of both the EGFR homodimer and the EGFR-HER2 heterodimer were destabilized when the EGFR lost EGF (a specific ligand of EGFR) [15]. We therefore conducted MD simulations of the HER2 mutants complexed with ligand-free or ligand-bound EGFR. These simulations showed that the surface area buried in the dimer interface of the EGFR-HER2 WT heterodimer significantly decreased under the ligand-free condition compared to the ligand-bound condition (Fig. 4a and b). Interestingly, the decrease in the buried surface area that resulted from removing EGF was not statistically significant in the EGFR-HER2 E401G and EGFR-HER2 S310F heterodimers (Fig. 4a and b), consistent with the results of the phosphorylation levels of HER2 and EGFR. Whereas the mean simulation structure of HER2 WT complexed with ligand-free EGFR showed a remarkable gap in the dimer interface, the gap tended to be closed in the structures of the HER2 E401G and S310F mutants. (Fig. 5 and Fig. S3). On the other hand, MD simulations of the HER2 homodimer suggested that there are no statistically significant differences in the surface areas buried in the dimer interface between WT and the two mutants, whereas these mutations appear to stabilize the dimer interface (Fig. S4).

### HER2 p.(E401G) activates the MAPK pathway: A main downstream signaling pathway of the EGFR-HER2 heterodimer

**Fig. 6:**
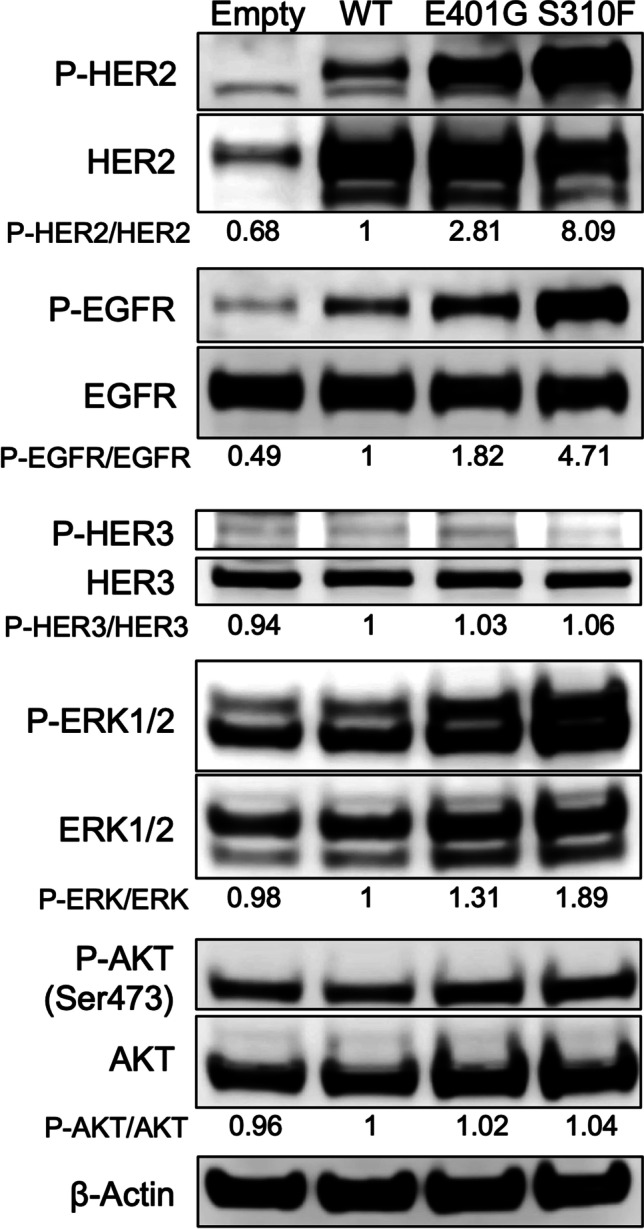
Phosphorylation of ERK and AKT in cells expressing *ERBB2* E401G. H460 cells were transiently transfected with *ERBB2* E401G, S310F or WT, cultured under serum-starved conditions, and exposed for three hours to serum-containing medium, after which whole-cell lysates were extracted, followed by Western blotting. Empty, empty vector; WT, wild type; P-, phosphorylated

Our simulation data showed that the activating mechanisms of *ERBB2* E401G and S310F were related to the EGFR-HER2 heterodimer. The dimerization partner appears to be an important determinant of signaling activity. The two main pathways activated by HER family dimers are the mitogen-activated protein kinase (MAPK) pathway and the phosphatidylinositol 3-kinase (PI3K)-AKT pathway [37]. The EGFR-HER2 heterodimer and the HER2 homodimer are mainly related to the MAPK pathway (RAS-RAF-MEK-ERK pathway), whereas the HER2-HER3 heterodimer is related to the PI3K-AKT pathway [38]. To confirm whether the MAPK pathway is truly activated in cells expressing *ERBB2* E401G and S310F, we examined the phosphorylation of downstream signaling pathway proteins in H460 cells. We found that the phosphorylation of ERK was elevated in cells expressing *ERBB2* E401G and S310F, whereas that of AKT was unchanged (Fig. 6), consistent with our simulation data.

### Biological effects of *ERBB2* E401G

**Fig. 7:**
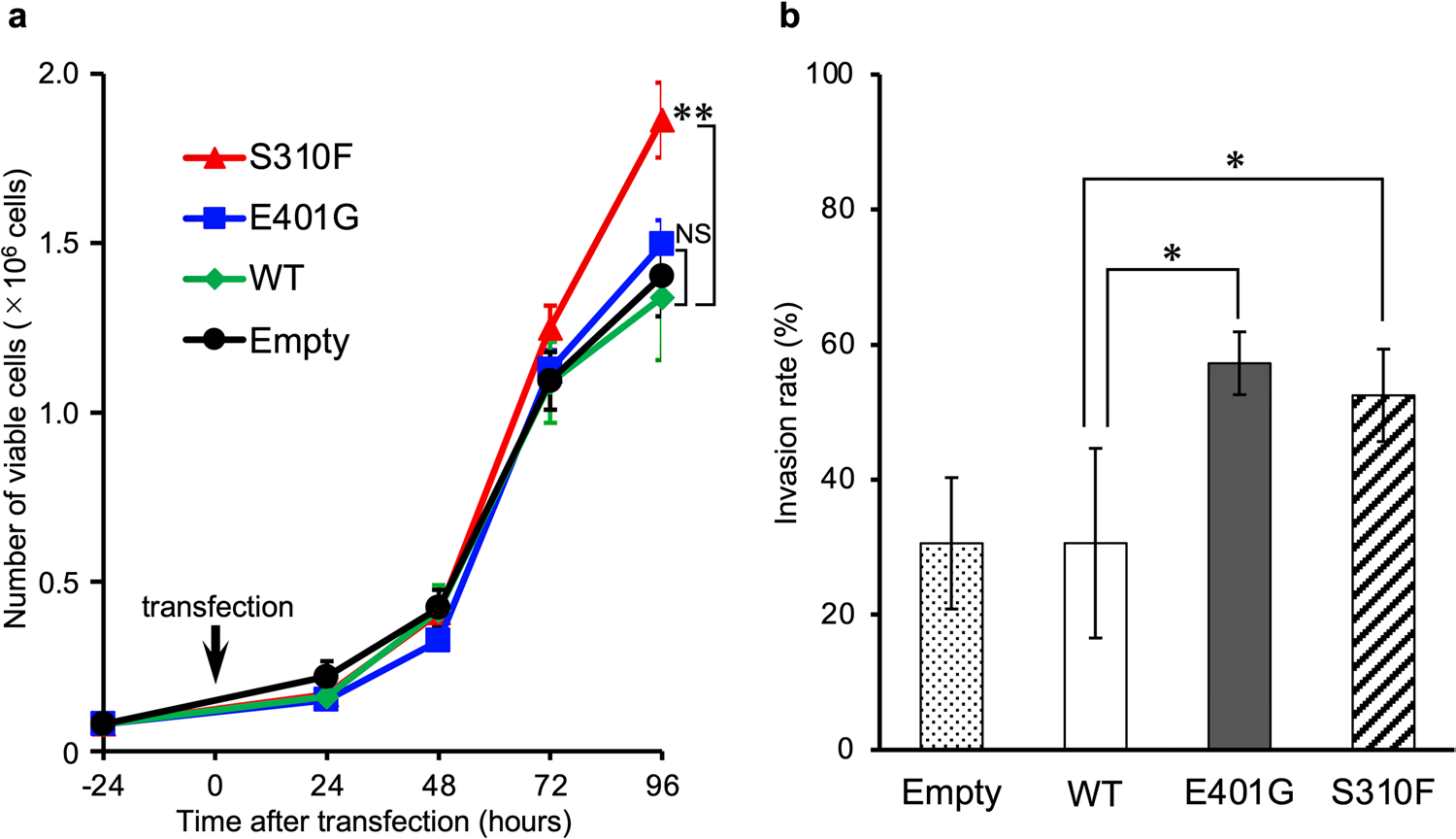
In vitro evaluation of the biological function of *ERBB2* E401G using H460 cells. The cells were transiently transfected with *ERBB2* E401G, S310F or WT, after which the number of viable cells for the indicated period (**a)** and the invasion rate 24 h after seeding (**b)** were assessed. The invasive capacities of cells expressing E401G or S310F were significantly greater than those of cells expressing WT. The results are presented as mean values of three independent experiments. Error bars, SD. Differences between groups were evaluated using one-way ANOVA and Dunnett multiple comparison tests. *p < 0.05 and **p < 0.01 vs. wild type. NS, not significant; empty, empty vector; WT, wild type

To examine the biologic effects of *ERBB2* E401G in cancer cells, we evaluated the proliferative and invasive capacities of H460 cells. We found that cells expressing *ERBB2* S310F exhibited a significantly higher proliferation rate than cells expressing *ERBB2* WT, whereas cells expressing *ERBB2* E401G did not (Fig. 7a). Conversely, we found that the invasive capacity of cells expressing either *ERBB2* E401G or S310F was significantly higher than that of cells expressing *ERBB2* WT (Fig. 7b). Additionally, we conducted a soft agar colony formation assay with stably transfected NIH3T3 cells using the same vectors, and found that NIH3T3 cells expressing *ERBB2* E401G or S310F did not form apparent colonies (Fig. S5).

**Fig. 8:**
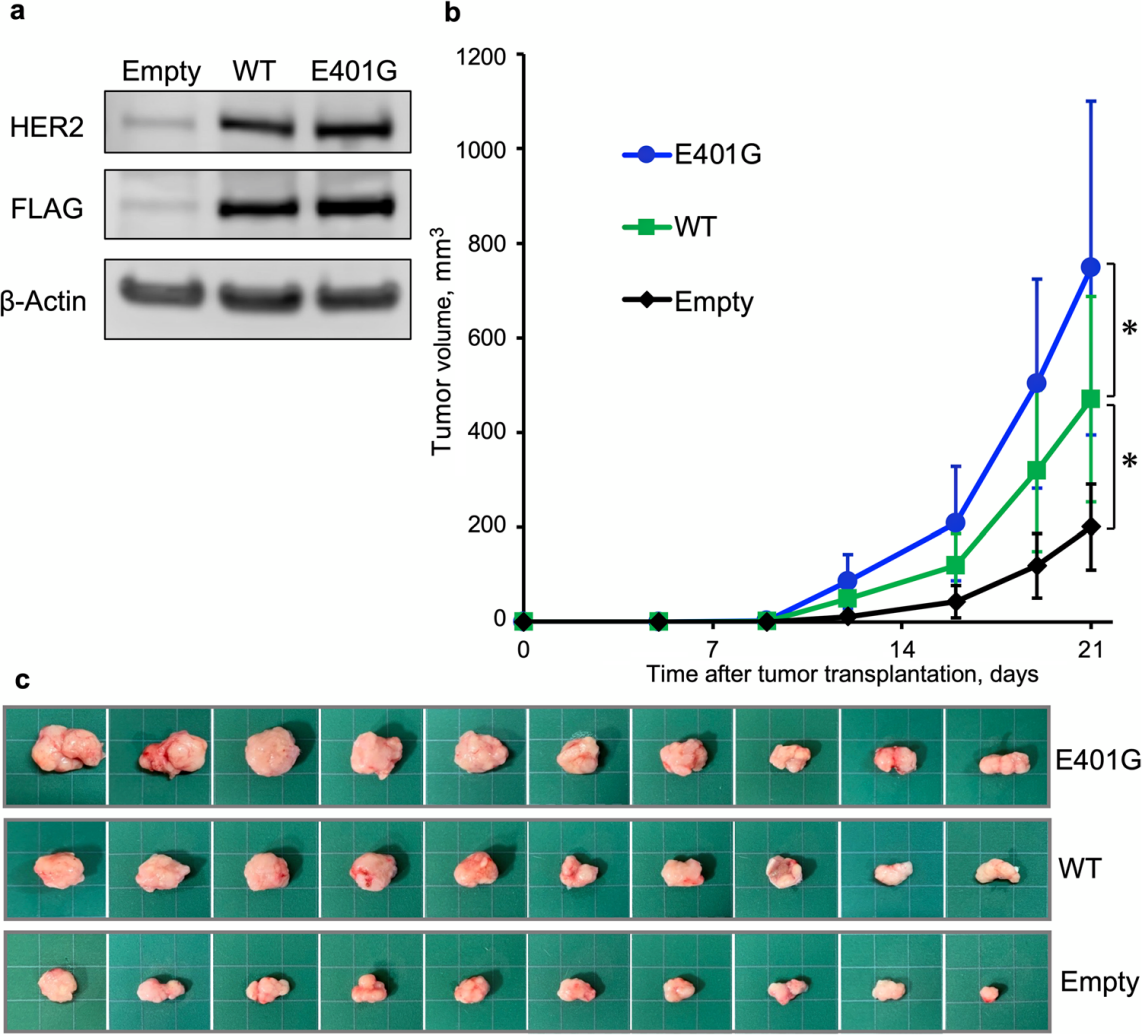
Tumor formation by *ERBB2* E401G expressing cells in a xenograft mouse model. **a** Stable transformants of H460 cells, carrying *ERBB2* WT, *HER2* E401G or vector alone (empty), were established. **b** H460 cells stably expressing *ERBB2* were injected into the right dorsal flanks of BRJ mice. Tumor volumes were measured on the indicated days after transplantation (n = 10 mice in each group). Differences between groups were evaluated using one-way ANOVA and Dunnett multiple comparison tests. *p < 0.05 vs. wild type. **c** Appearance of tumors at the time when the mice were sacrificed (21st day after transplantation). Empty, empty vector; WT, wild type

To examine tumor forming capacity in vivo, we constructed H460 cells that stably express *ERBB2* (Fig. 8a) and assessed tumor growth after subcutaneous inoculation of these cells into mice. On the 21st day after transplantation, the tumor growth of cells expressing *ERBB2* E401G was found to be significantly increased compared to that of cells expressing *ERBB2* WT (Fig. 8b and c).

## Discussion

We found, by assuming pathogenicity with multiple computational predictive algorithms, that a particular VUS, *ERBB2* E401G, has a biological effect on oncogenicity. We used multiple approaches, including in vitro cell and in vivo animal experiments. Additional signal transduction and in silico MD simulation revealed molecular mechanisms underlying the biological effects, in which *ERBB2* E401G causes gain of function via stabilization of the HER2-EGFR heterodimer. On the basis of these results, patients with *ERBB2* amplification and a missense variant E401G are expected to benefit from chemotherapy using anti-HER2 antibodies, and so they were recruited into a basket trial of trastuzumab and pertuzumab.

*ERBB2* E401G is a novel variant that has not been registered in principal databases of disease-associated variants, such as COSMIC and ClinVar. Thus, it was considered to be a VUS. According to the guidelines for interpretation of variants in cancer, a VUS should not have been reported at significant allele frequencies (the cut-off recommended by the working group is 0.01) in the general population to distinguish it from a SNP [4]. *ERBB2* E401G has not been observed in any general population database, including the 1000 Genomes Project Database, ExAC and dbSNP. This supports the conclusion that it is unlikely to be a SNP. Multiple computational tools are recommended for predicting the pathogenicity of variants since each different tool has its own strengths and weaknesses [39]. In the present study, several prediction tools suggested deleterious effects of *ERBB2* E401G. In the gene panel test in the present case, besides *ERBB2* E401G, short variants of *TP53*, *EZH2*, *BAP1* and *FH*, and homozygous deletions of *CDKN2A* and *FANCC* were co-existent (Fig. 1c), all of which are pathogenic variants and potential cancer driver genes. Although it is difficult to establish which variants play a central role in the development of the cancer in this case, we speculated that *ERBB2* E401G, which has a high allele fraction with amplification, may play a major role in this CUP involving mainly abdominal lymph nodes and subcutaneous tumor growth. Moreover, in contrast to *ERBB2* kinase domain variants, there are few studies dealing with the mechanisms by which *ERBB2* ECD variants activate certain cellular processes related to cancer. Therefore, we considered it valuable to perform functional analyses of *ERBB2* E401G to assess its oncogenicity and its mechanism of activation.

Previously, two distinct mechanisms have been reported by which *ERBB2* ECD variants activate certain cellular processes associated with cancer, i.e., the formation of disulfide-linked dimers and an increase in C-terminal phosphorylation [11]. In the present study, E401G did not show any difference in disulfide-linked dimers relative to the level of the dimers formed with WT protein but, instead, showed increased C-terminal phosphorylation of HER2, similar to S310F. These results suggest that E401G exhibits functional properties that are similar to those of S310F. Although the mechanisms of increased C-terminal phosphorylation of *ERBB2* ECD variants such as S310F are not well understood, it has been reported that the HER2 S310F mutant preferentially forms an active heterodimer with EGFR, which was revealed by different reactivity to anti-HER2 or anti-EGFR antibodies and a single molecule interaction analysis using TIRF microscopy [13]. In general, HER family proteins are phosphorylated through trans-phosphorylation via dimerization with another HER family member, leading to recruitment and activation of downstream proteins [31, 32]. Our results showed that cells expressing *ERBB2* E401G and S310F increased the C-terminal phosphorylation of HER2 and EGFR, but not that of HER3, suggesting that heterodimerization with EGFR, homodimerization of HER2, or both may be related with the activation of these variants. To further clarify these mechanisms, we conducted MD simulation, which revealed that HER2 E401G and S310F mutants stabilize the EGFR-HER2 heterodimer in the ligand-free condition. In addition, we found that the MAPK pathway, a downstream signaling pathway of the EGFR-HER2 heterodimer, was activated in both E401G and S310F cells. These results suggest that the activating mechanisms of *ERBB2* E401G and S310F act via EGFR-HER2 heterodimer formation in a ligand-independent manner.

Activating mutations of *ERBB2*, which are mainly observed in the tyrosine kinase domain, lead to increased cell proliferation in vitro, rapid tumor growth in vivo, and sensitivity to HER2 targeted therapy, indicating their role as driver mutations [7, 8, 12]. In our study, exogenous expression of *ERBB2* E401G in H460 cells led to an increased invasive capacity in vitro and an increased growth capacity in a mouse model compared to those of *ERBB2* WT transfected cells. Overexpression of HER2 is known to function as an oncogenic driver [40], and some tumors exhibit both *ERBB2* variants and amplification [12]. Our patient exhibited *ERBB2* amplification in addition to an *ERBB2* E401G variant, suggesting that both *ERBB2* mutation and amplification has led to tumor development.

As for the interpretation of variants, the AMP/ASCO guidelines [4] require “multiple lines of reported evidence” for Tier II, Variants of potential Clinical Significance. As yet, it is difficult for *ERBB2* E401G to be categorized as Tier II. According to the ACMG guidelines [39], when the data is judged as PS3, i.e., well-established in vitro or in vivo functional studies supportive of a damaging effect, it is classified as Likely Pathogenic. With regard to the PS3 criteria, the Clinical Genome Resource (ClinGen) Sequence Variant Interpretation (SVI) Working Group has proposed a four-step provisional framework for determining the appropriate strength of evidence [41], and on that basis our data are classified as PS3 supporting.

In this paper, we report an integrated approach to the functional analysis of VUS that uses conventional in vitro and in vivo data combined with computational simulation analyses. To effectively use cancer panel tests, rapid and precise analysis of variants detected in the tests is required for selecting the appropriate treatment strategy. Although the workflow needs to be further improved so that it depends more closely on the characteristics of each variant, the integrated approach presented here can facilitate the functional analysis of VUS detected in a cancer gene panel test.

## Conclusions

**Fig. 9:**
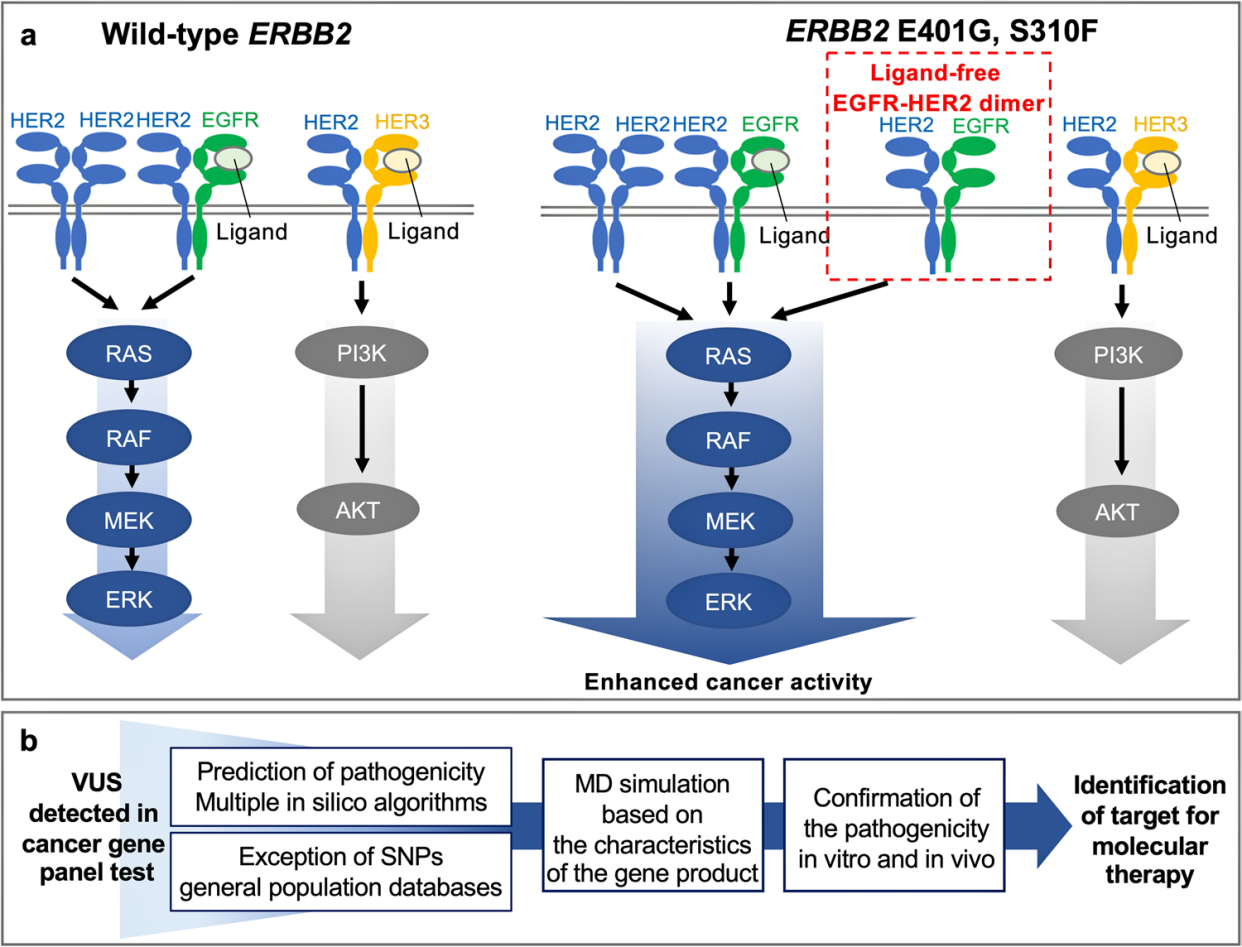
Summary of our study. **a** Schematic view of functional changes of *ERBB2* E401G and S310F mutants. *ERBB2* E401G and S310F mutants can stabilize ligand-free EGFR-HER2 heterodimers and activate the downstream MAPK pathway more efficiently than WT. These changes cause enhanced cancer activity, including increased invasive and proliferative capacities. **b** Workflow of functional analysis of VUS. After narrowing down the likely pathogenic variants using in silico prediction algorithms and general population databases, a combination of MD simulation analysis and in vitro and in vivo studies can lead to efficient functional analyses and identification of targets for molecular therapy

With the ongoing implementation of NGS-based assays in clinical practice, the number of VUS will inevitably increase. Therefore, it is essential to establish efficient functional analysis methods in conjunction with in silico simulation assays that depend on gene characteristics. Using an integrated in vitro, in vivo and in silico analysis, we found that *ERBB2* E401G, a novel VUS of ECD III that was detected by a NGS panel test in a CUP patient, represents a gain-of-function variant. We also revealed new clues about the activating mechanisms of ECD variants related to elevated C-terminal phosphorylation: E401G and S310F mutants increase the contact area of ligand-free EGFR-HER2 heterodimers and stabilize them (Fig. 9a and b). Our results may have important implications for elucidating the activating mechanisms of *ERBB2* ECD variants and for defining a model workflow for analyzing VUS detected by cancer gene panel tests.

## Supplementary Information

ESM 1 (PDF 1.68 mb)

## Data Availability

All data related to this study are included in this article and its supplementary information files. The datasets used and analyzed during the current study are available from the corresponding author upon reasonable request.

## Abbreviations

HER: Human epidermal growth factor receptor
VUS: Variants of unknown significance
AMP: Association for Molecular Pathology
NGS: Next-generation sequencing
ECD: Extracellular domain
CUP: Cancer of unknown primary origin
MD: Molecular dynamics
ddPCR: Droplet digital PCR
WT: Wild type
BRJ: Balb/c Rag-2^−/−^ Jak3^−/−^
SD: Standard deviation
CT: Computed-tomography
FDG: Fluorodeoxyglucose
IHC: Immunohistochemistry
CK: Cytokeratin
FISH: Fluorescence in situ hybridization
MAPK: Mitogen-activated protein kinase
PI3K: Phosphatidylinositol 3-kinase
