# Supplementary material for: Integrated approach to functional analysis of an ERBB2 variant of unknown significance detected by a cancer gene panel test
Source: Cell Oncol (Dordr). 2022 Jan 8;45(1):121–34. doi: 10.1007/s13402-021-00656-3 (PMC8881279; doi:10.1007/s13402-021-00656-3)
Supplement: Supplementary file 1 — (PDF 1.68 mb) [file 13402_2021_656_MOESM1_ESM.pdf]

Cellular Oncology

Integrated approach to functional analysis of an *ERBB2* variant of unknown significance detected by a cancer gene panel test

Yohei Harada[1], Akemi Sato[2], Mitsugu Araki[3], Shigeyuki Matsumoto[3,4], Yuta Isaka[5], Yukari Sagae[3], Tomonori Abe[1], Yasuko Aoyagi[6], Eisaburo Sueoka[2], Yasushi Okuno[3,4,5], Shinya Kimura[1] and Naoko Sueoka-Aragane[1]*

[1] Division of Hematology, Respiratory Medicine and Oncology, Department of Internal Medicine, Faculty of Medicine, Saga University, 5-1-1 Nabeshima, Saga 849-8501, Japan.

[2] Department of Clinical Laboratory Medicine, Faculty of Medicine, Saga University, 5-1-1 Nabeshima, Saga 849-8501, Japan.

[3] Graduate School of Medicine, Kyoto University, 53 Shogoin-Kawaharacho, Sakyo-ku, Kyoto 606-8507, Japan.

[4] Medical Sciences Innovation Hub Program, RIKEN Cluster for Science, Technology and Innovation Hub, 1-7-22 Suehiro-cho, Tsurumi-ku, Yokohama City, Kanagawa, 230-0045, Japan.

[5] AI-driven Drug Discovery Collaborative Unit, RIKEN Center for Computational Science (R-CCS), HPC- and AI-driven Drug Development Platform Division, 7-1-26 Minatojimaminamimachi Chuo-ku, Kobe City, Hyogo 650-0047, Japan.

[6] Department of Precision Cancer Medicine, Center for Innovative Cancer Treatment, Tokyo Medical and Dental University, 1-5-45 Yushima, Bunkyo-ku, Tokyo 113-8510, Japan.

*Naoko Sueoka-Aragane　E-mail: sueokan@cc.saga-u.ac.jp

Fig. S1 HER2 and FLAG expression levels after transfection of *ERBB2*-expression vectors into H460 cells and NIH3T3 cells. Transiently transfected H460 cells and NIH3T3 cells were grown in complete medium for 48 hours, after which whole-cell lysates were extracted.

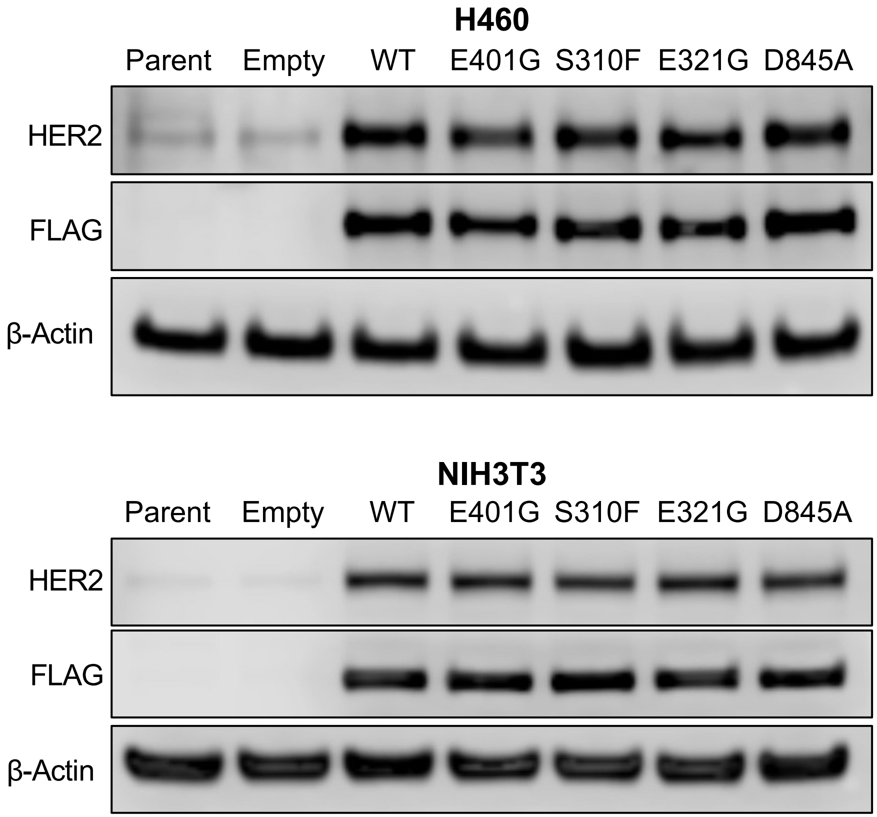

Fig. S2 Stability of the dimer interface of the “back-to-back” and “back-to-head” HER2 dimer models in MD simulation. **a** Initial structure models of the “back-to-back” dimer [PDB code: 3NJP (the EGFR homodimer bound to EGF)] and the “back-to-head” dimer [PDB code: 3WLW (the HER2 homodimer)]. Subunits A and B in the dimer and EGF are depicted by green, cyan, and orange ribbon diagrams, respectively. EGFR residues 240-309 and HER2 residues 268-338 in domain II are highlighted in magenta. **b** Root-mean-square deviation (RMSD) of the backbone Ca atoms from the initial structure for the “back-to-back” EGFR-HER2 heterodimer bound to EGF, the “back-to-back” EGFR-HER2 heterodimer, the “back-to-back” HER2-HER2 homodimer, the “back-to-head” EGFR-HER2 heterodimer, and the “back-to-head” HER2-HER2 homodimer models. Time evolutions of RMSD during three independent simulations of 1000 ns (black, red, and green) are shown for each structure model. Overall structures of the “back-to-back” dimer models were stably maintained during 1000 ns simulations whereas those of the “back-to-head” models were relatively unstable even in the HER2-HER2 homodimer.

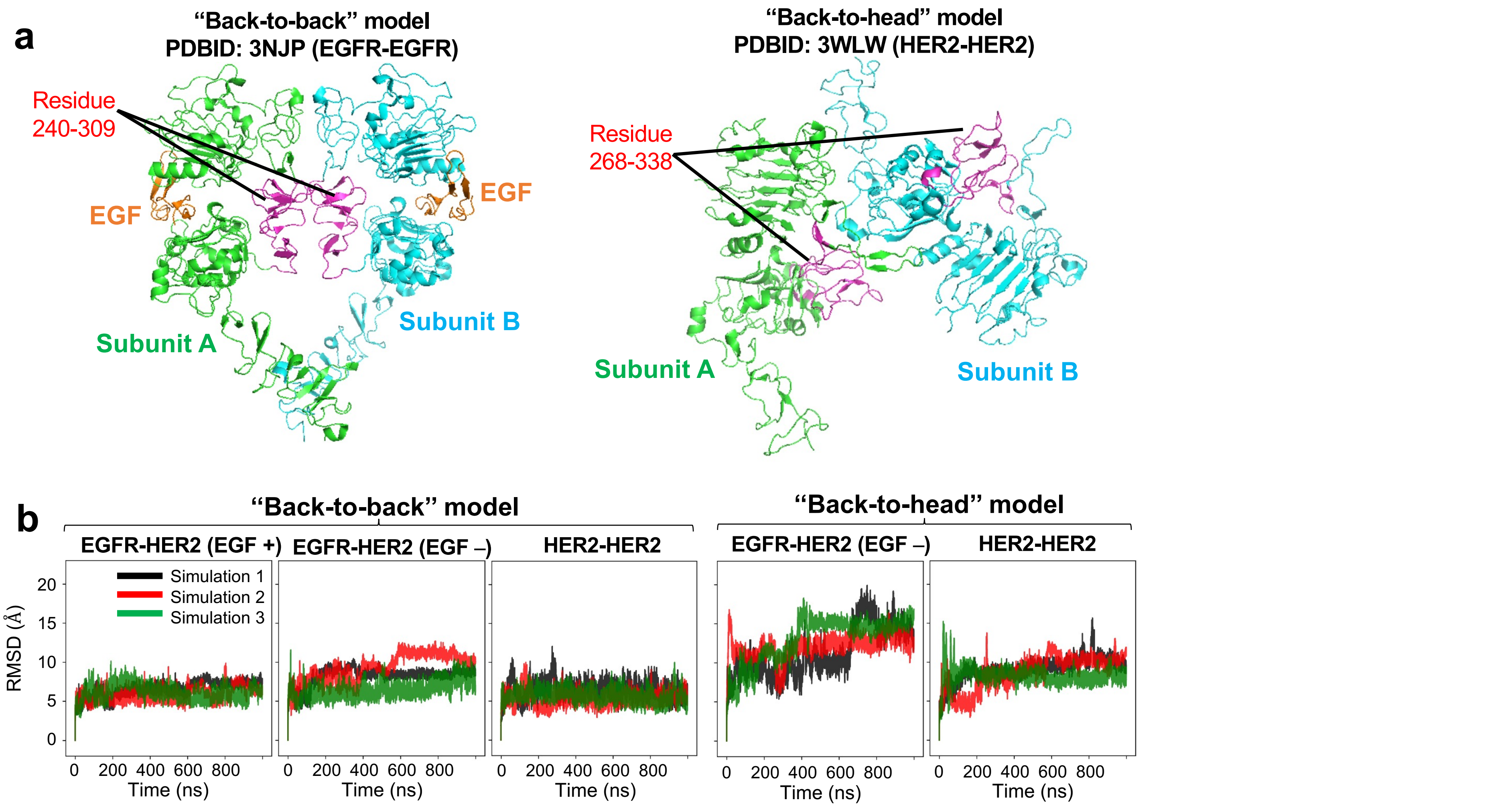

Fig. S3 Comparison of MD simulation structures of (**a**) the EGFR-HER2 S310F and (**b**) the EGFR-HER2 E401G dimers with those of the EGFR-HER2 WT dimer. The protein backbones (domain I-III) of the EGFR-HER2 S310F (E401G), EGFR-HER2 WT, and EGFR/EGF-HER2 WT dimers are represented by ribbon diagrams, after the EGFR subunits in these dimers were structurally aligned. The side chains of S/F310 and E/G401 are depicted as spheres.

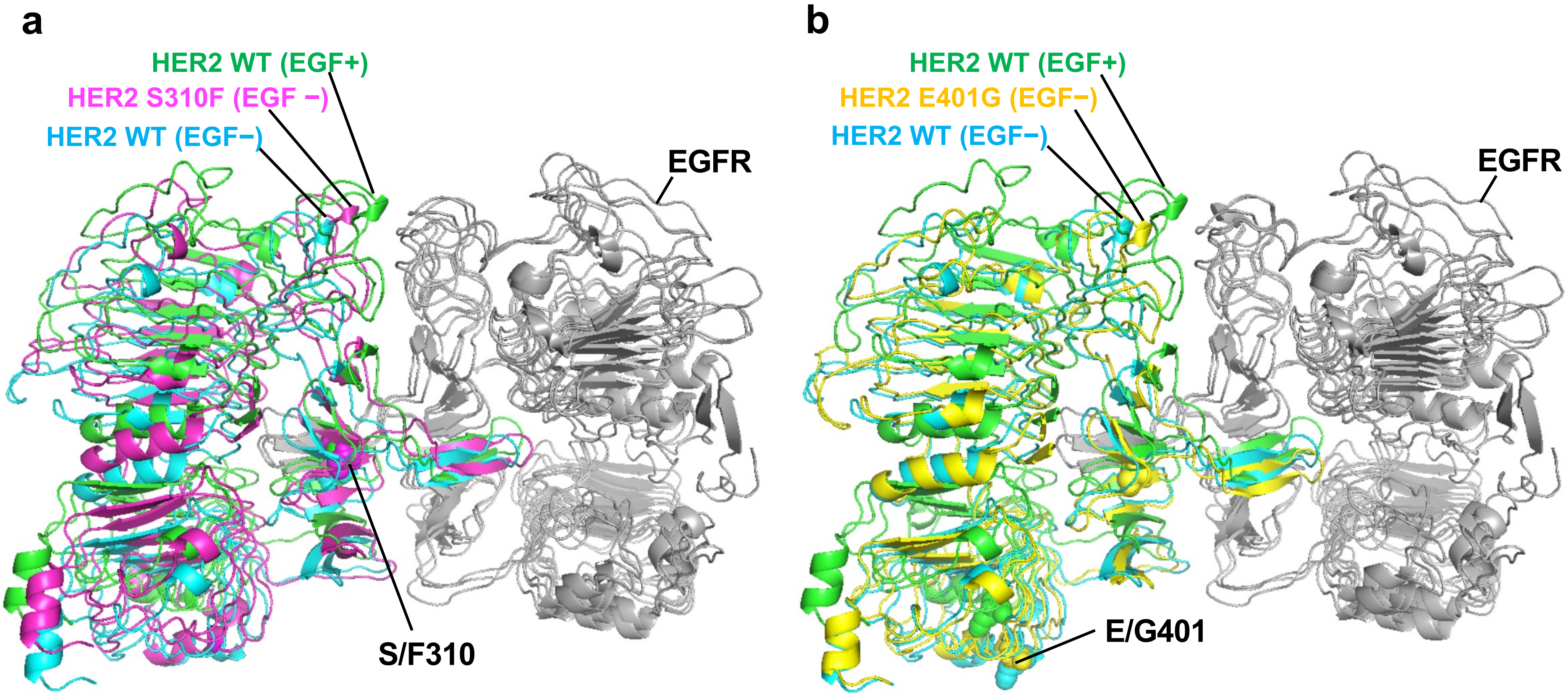

Fig. S4 Stability of the dimer interface of the HER2-HER2 homodimer in MD simulation. **a** The surface area buried in the dimer interface during five independent simulations of 1000 ns (black, red, green, blue, and orange). Time-dependent transition of the buried area is plotted with thin lines along with a 10 ns window average of (thick lines). **b** The buried area averaged across trajectories of 500–1000 ns extracted from the five simulations. The difference in the buried area between HER2 WT and its E401G or S310F mutant was evaluated with a one-way ANOVA. Although these mutations appear to stabilize the dimer interface, no statistically significant differences were observed.

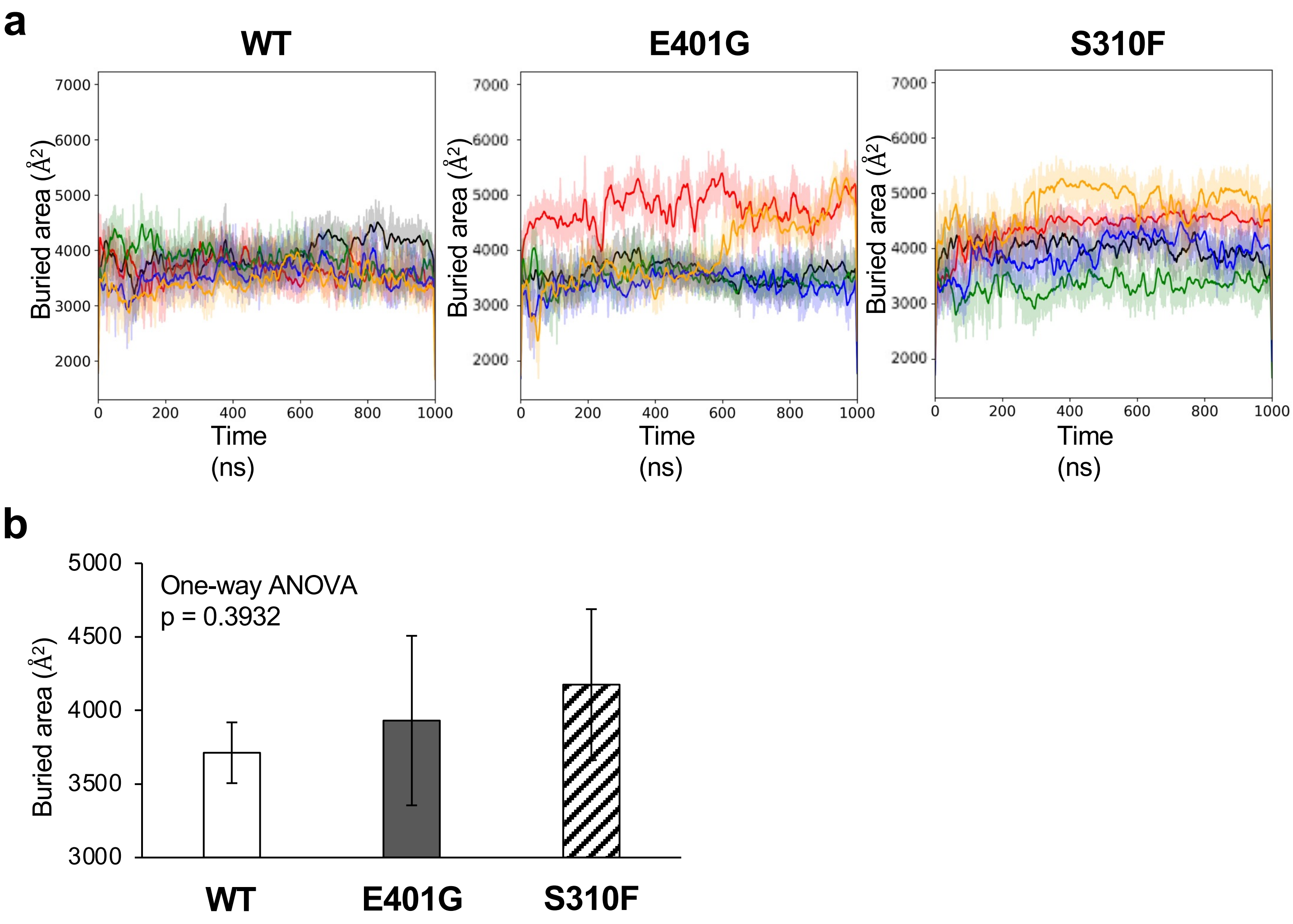

Fig. S5 Soft agar colony formation assay using stably transformed NIH3T3 cells. **a** Stably transformed NIH3T3 cells with wild type *ERBB2*, E401G *ERBB2*, S310F *ERBB2*, or vector alone (empty) were established by a selection procedure using G418 after transfection. **b** A549 cells (human lung cancer cell line) were used as a positive control. Even S310F, an activating mutation of ECD, did not form a robust colony in our experiments.

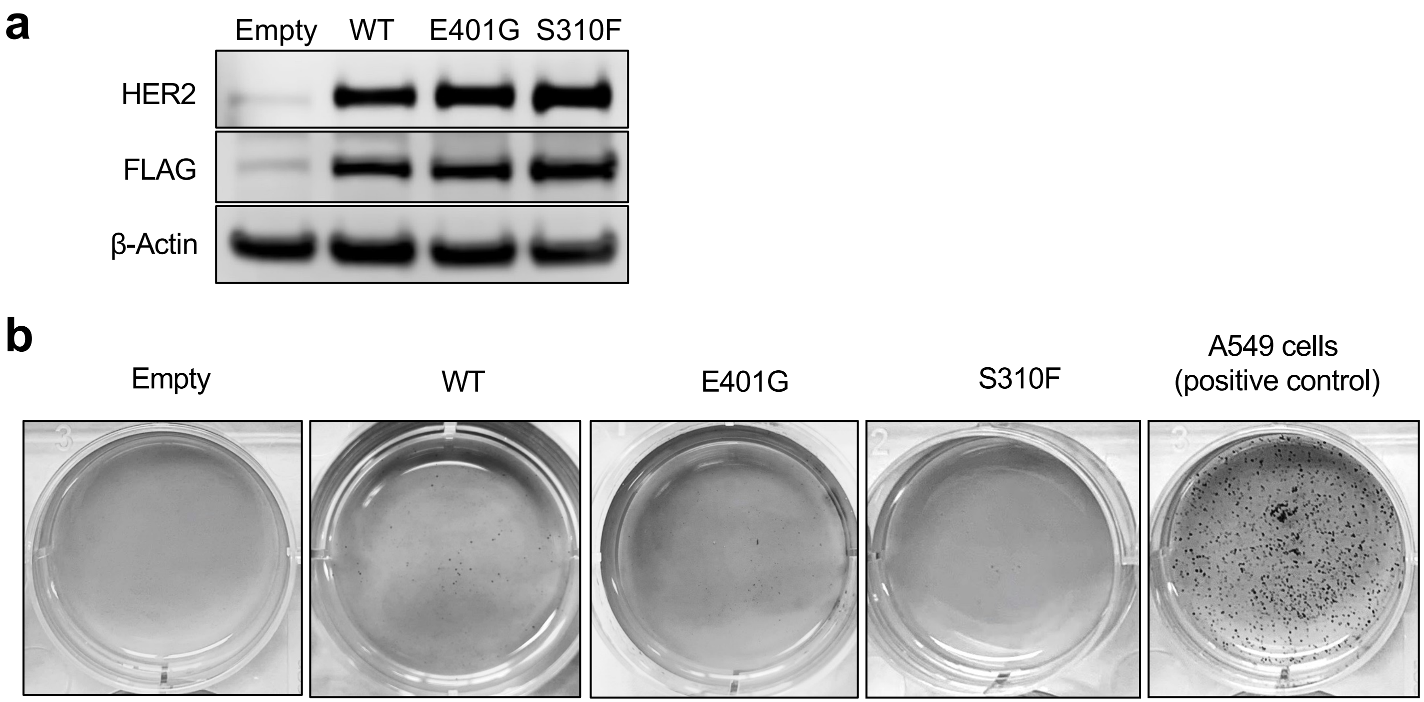

Supplementary methods

Soft Agar colony formation assay

Soft agar assays were performed as reported previously [11]. Briefly, 1.5×10^3 cells were suspended in media containing 0.3% SeaPlaque GTG agarose (Cambrex, Rockland, ME, USA) and plated on a bottom layer of media containing 0.6% NuSieve™ 3:1 agarose (Lonza, Rockland, ME, USA) in a six-well plate. Plates were incubated at 37℃ for 2 weeks, after which imaging was conducted. The plates were photographed after 2 hr staining with MTT.
